# Closure of post-laryngectomy pharyngocutaneous fistulae

**DOI:** 10.1186/1758-3284-3-29

**Published:** 2011-05-26

**Authors:** Isaac A Bohannon, William R Carroll, J Scott Magnuson, Eben L Rosenthal

**Affiliations:** 1 University of Washington, Puget Sound Veterans Administration Health System Department of Otolaryngology Head and Neck Surgery 1660 S Columbian Way, Mail Stop: S-112OTO, Seattle, WA, 98108, USA; 2 University of Alabama at Birmingham, Department of Surgery, Division of Otolaryngology Head and Neck Surgery BDB 1530 3rd Ave South, Birmingham, AL, 35249-0012, USA

**Keywords:** Laryngectomy, Free flap, Pharyngocutaneous fistula, Head and neck cancer, Wound reconstruction, Hypothyroidism, Salvage

## Abstract

**Background:**

Closure of salvage laryngectomy defects with vascularized tissue remains controversial.

**Methods:**

We evaluate outcomes in patients who required repair of a fistula after attempted primary closure of salvage laryngectomy defect and assess risk factors for persistent fistula. Between 2001 and 2010, 20 patients were treated for pharyngocutaneous fistulae after primary closure of a salvage laryngectomy. All patients required free flap repair for definitive fistula management.

**Results:**

Patients presented with fistulae from one to 18 months in duration; median time to closure was seven days. Radial forearm free flap was used in 86% of patients. With free flap alone 50% of patients achieved fistula closure. Additional procedures improved closure rate to 85%. Recipient vessels were used in the neck in 54.5%, compared to internal mammary vessels in 45.5%. Hypothyroidism was identified as a risk factor for persistent fistula (p = 0.01). Chronic steroid use (p = 0.08) did not reach significance as a risk factor for fistula closure. Gastroesophageal reflux disease was newly diagnosed or noted as a comorbidity in 14 patients (70%) in this study. It did not reach statistical significance as a risk factor in refistulization (p = 0.12). Complications included leak, carotid blowout, infection, free flap loss, and late refistulization. Overall flap failure in this study was 4.5%.

**Conclusions:**

Delayed secondary repair of pharygocutaneous fistulas after salvage laryngectomy is associated with a higher complication rate and poor success rates compared to use of vascularized tissue at the time of salvage laryngectomy. Prolonged wound healing in these patients is associated with hypothyroidism.

## Introduction

There are almost 12,000 newly diagnosed cases of laryngeal cancer every year in the United States [1]. The primary advantage of chemoradiation protocols for squamous cell carcinoma of the head and neck is organ preservation--with the potential for preservation of natural speech and swallowing function. Unfortunately, structural preservation does not equate to normal function. While an adequate voice has been reasonably easy to maintain, adequate swallowing has not [2-4]. Current management trends have favored radiation or chemoradiation over surgical options in the spirit of that goal. As a result, there have been a growing number of patients presenting with recurrent disease requiring salvage laryngectomy. Closure of salvage laryngectomy defects after chemoradiation is associated with a 3%-65% [5,6] wound complication rate, with the most common and significant problem being fistula formation.

Although it is well known that concomitant chemoradiotherapy intensifies the clinical side effects, the complex pathophysiology must continue to be investigated. Tissue in the field of radiation treatment undergoes multiple types of injury, most commonly referred to as radiation-induced fibroatrophy [7]. In brief, the vascular endothelial cells experience oxidative injury, which leads to the recruitment of inflammatory cells, and a cascade of cytokines. Fibroblasts and myofibroblasts secrete excessive and altered extracellular matrix. The capillary network is damaged both acutely and chronically. This impacts the normal surrounding mucosa in addition to the adjacent tumor. Thus, the mucosa remaining after laryngectomy is friable and poorly vascularized.

It has been recently been shown that introduction of vascularized tissue at the time of initial pharyngeal closure reduces the fistula rate and need for operative intervention [5,8,9]. However, primary closure of salvage laryngectomy defects remains the standard-of-care at most institutions. Although many post-salvage laryngectomy fistulae can be managed conservatively, poor tissue vascularity, malnutrition, and hypothyroidism [6,10] can lead to chronic fistula formation which requires aggressive reconstruction to definitively re-establish an oral diet.

Our strategy to secondarily repair post-laryngectomy defects has evolved significantly over the past decade as a result of experience with these complex wounds. We present our experience and the reconstructive strategy refined from this experience as applied to pharyngocutaneous defects after salvage laryngectomy in a review of 20 patients who underwent secondary fistula repair.

## Patients and Methods

This retrospective study reviewed over 1300 free flap reconstructions performed between 2001 and 2010. We identified 20 patients who required reconstruction of their pharyngocutaneous fistula after salvage laryngectomy with primary closure. Patients were primarily treated at our institution, as well as referred from the surrounding region after fistula development. After Institutional Review Board approval all data was gathered from patient charts and electronic medical record.

Fistulae were categorized as wound dehiscence resulting in fistula or stricture-associated fistula, based on the senior author's pan endoscopy exam. Patients with tracheoesophageal puncture-related fistula were not included in this study. Detailed demographic information was obtained including prior surgeries, radiation treatment, duration of fistula, weight loss, and comorbidities. Endocrine status was assessed by thyroid stimulating hormone (TSH) levels, when deemed necessary. Nutritional status based on albumin and prealbumin levels were collected on a case-by-case basis. Comorbidities were obtained based on patient history, medication list, and/or laboratory data. All patients eventually required free flap reconstruction. Free flap is defined as free tissue transfer from another location in the body with microvascular anastomosis of donor vasculature to selected recipient vessels in the neck. Information on the type of free flap used, location of vessel anastomosis, presence of internal dopplers, length of hospital stay, and complications was obtained. In the post-operative period barium swallow was used to assess for fistula closure. Pedicled flaps were used in certain patients, defined as tissue flaps which are rotated on a pedicled blood supply, such as a pectoralis muscular or musculocutaneous flap. Local flap use was also noted in the patients' course of treatment. Local flaps were defined as small cutaneous tissue rearrangements directly adjacent to the fistula. Patients were assessed for type of diet, method of communication, and weight gain during their follow-up. Patients were followed at least until fistula closure.

Statistical analysis was performed to examine patterns that predisposed patients to recurrent fistula. Continuous variables were analyzed with t-test. Categorical variables were analyzed with Fisher's exact test. For all analyses, p value < 0.05 was deemed statistically significant.

## Results

Free flaps were performed on all patients to obtain definitive closure. There were 22 free flaps performed in 20 patients. Table 1 describes each patient's course of treatment. Approximately 86% of patients had radial forearm flap (RFFF) reconstruction, but other flaps included anteriolateral thigh (ALT) and rectus free flaps.

**Table 1:** Comorbidity, Reconstruction Technique, Complications, and Associated Time to Fistula Closure

Patient	Gender	Age	Comorbidities	Duration of Fistula (months)	Flap(s)	Defect	Survival	Vessels (A/V)	Complications	Time to Closure (days)	Other
1	M	69	None	5	RFFF	folded	Yes	EC/EJ	None	7	N
2	M	65	DM, HT, GERD	3	RFFF	patch	Yes	EC/IJ	Contained leak	7	N
3	F	47	DM,GERD	3	RFFF	folded	Yes	EC/IJ	None	6	N
4	F	67	HT, GERD	13	RFFF	tubed	Yes	F/EJ	None	10	N
5	F	66	None	1	RFFF	patch	Yes	F/IJ	None	5	N
6	M	59	GERD	4	RFFF	folded	Yes	F/EJ	None	40	N
7	M	66	HT, GERD	8	RFFF	onlay	Yes	F/IJ	Persistent leak	39	Y
8	F	69	HT, GERD	4	RFFF	folded	Yes	F/IJ	None	7	N
9	F	49	DM, Hepatitis	7	RFFF	patch	Yes	F/IJ	None	7	N
10	M	53	GERD	13	Rectus	patch	Yes	IM/IM	None	9	N
11	M	70	HT	5	Rectus, RFFF	tubed, patch	Yes	IM/IM, IM/IM	Diverting fistula, hematoma	35	Y
12	M	53	None	1	RFFF	patch	Yes	IM/IM	None	6	N
13	F	67	GERD	2	RFFF	onlay	Yes	IM/IM	Contained leak	6	N
14	M	66	HT, GERD	5	ALT	patch	Yes	IM/IM	Refistulized	90	Y
15	M	64	DM, HT, Steroid, GERD	4	RFFF	onlay	Yes	IM/IM	PNA, Refistulized	53	Y
16	M	68	Steroid, HT, GERD	4	RFFF	folded	No	ST/EJ	CC blowout, lig, Pec, persistent leak	Never	Y
17	F	75	HT, GERD	7	RFFF	folded	Yes	EC/IJ	None	7	N
18	M	54	DM, HT, GERD	1	RFFF	folded	Yes	F/IJ	Late pharyngocarotid fistula, CC stent, Pec, CC lig, Refistulized	Never	Y
19	M	62	HT, GERD	9	RFFF, RFFF	folded, onlay	Yes	IM/IM, IM/IM	Neck and chest infection, refistulized	Never	Y
20	M	49	None	18	RFFF/Pec	patch	Yes	IM	Contained leak	7	N

M, male; F, female; DM, diabetes mellitus; GERD, gastroesophageal reflux disease; HT, hypothyroidism; Steroid, chronic steroid use; RFFF, radial forearm free flap; ALT, anterolateral thigh free flap; Rectus, rectus abdominis free flap; Pec; pectoralis major pedicled flap; CC, common carotid; EC, external carotid; EJ, external jugular; IJ, internal jugular; IM, internal mammary; ST, superior thyroid; A, artery; V, vein; PNA, pneumonia; Other = additional procedures performed to facilitate fistula closure. Y = yes. N = no.

Recipient vessel choice was managed on a case-by-case basis, dependent on the patients' previous neck dissection status, quality of neck tissue on physical exam, and experience of the senior author. Internal mammary vessels (both artery and vein) were chosen as the recipient vessels in 45.5% of patients. Internal mammary vessels were favored in the later years of this series. Of the 54.5% of patients in which neck vessels were used, most commonly used was the facial artery, followed by an unnamed branch of the external carotid system. The venous system chosen in the neck was most commonly the facial vein or a large internal jugular vein stump, followed by the external jugular vein, and an end-to-side internal jugular vein anastomosis.

The fistula closure rate with the initial free flap procedure was 50%. Ten patients (50%) had a leak on barium swallow or refistulized requiring additional procedures. After a second free flap (Table 1, patient 11), simultaneous pectoralis pedicled flap (Table 1, patient 20), or additional local flaps (Table 1, patients 7, 14, 15) an overall closure rate of 85% was achieved. Patient 11 had planned, staged free flaps to obtain closure. He had a previous pectoralis flap before transfer to our institution. Six patients (17%) had mild leaks that were managed conservatively (Table 1, patients 2, 13, 20) or local flap closure (Table 1, patients 7, 14, 15). Three patients, even with additional local, pedicled, and free flaps, never attained fistula closure. Patients with persistent fistula were eventually lost to follow-up.

Interestingly, the duration of fistula prior to reconstruction was not found to be a statistically significant factor in the success of reconstruction. Indications for free flap included wound dehiscent fistula in 17 patients, and stricture-related fistula in only three patients. Perhaps due to the small numbers in the series, patients with wound dehiscent-related fistula (85%) were much more likely to achieve successful fistula closure than those deemed to have stricture-related fistula (15%). Of the three patients with stricture-related fistula, two of those three patients (66%) never achieved closure of their fistulae, compared to only one of 17 wound-dehiscent fistulae patients (6%). No patients in this series have required additional neopharyngeal dilations.

Complications were divided into early (during hospitalization) and late (post-discharge). There was one free flap loss in this series (4.5%). Other complications included infection (n = 1), hematoma (n = 1), and carotid blowout (n = 2). Of the two patients that suffered carotid blowout and subsequent ligation, both patients had prior neck dissections procedures and then underwent neck vessel anastomosis with subsequent persistent fistula/failure. One hematoma occurred due to bleeding from arterial feeding vessels in a rectus flap. Three patients had failure to close their fistula due to free flap loss, infection, or persistent late refistulization.

Hypothyroidism was a statistically significant comorbidity for refistulization (p = 0.01). Chronic steroid use (p = 0.08) did not reach significance as a risk factor for fistula closure. Gastroesophageal reflux disease was newly diagnosed or noted as a comorbidity in 14 patients (70%) in this study. However, it did not reach statistical significance as a risk factor in refistulization (p = 0.12). Three patients eventually required conversion of their gastrostomy tube to a gastro-jejunostomy tube to prevent reflux of gastric contents into the pharyngeal reconstruction.

The average time to fistula closure from the date of definitive fistula repair was 36 days and the median time to closure was seven days. Median hospital stay was 7 days (range 4-27 days) and more than half of patients were discharged on an oral diet on post-operative day (POD) seven. Median follow-up for patients after surgery was five months, but ranged from 2 to 55 months. During follow-up patients gained an average of 10 lbs, (p = 0.4).

Most patients (90%) used an electrolarynx for communication. Two patients underwent secondary tracheoesophageal puncture once the fistula was closed. Nearly 55% of patients returned to a regular or soft diet, 35% remained on liquid diet after fistula closure, and 10% were NPO using tube feeds, (Table 2).

**Table 2:** Types of postoperative communication and diet

Variable	All patients n (%)
	20 (100)
Communication method	
Electrolarynx	18 (90)
Tracheoesophageal puncture	2 (10)
Diet	
Regular/Soft	11 (55)
Liquid	7 (35)
NPO/Tube feeding only	2 (10)

NPO, *nil per os*.

In this series of patients 100% patients had prior radiation therapy, 25% had previous chemotherapy and 47% of patients had prior neck dissection. Of the 13 patients in the later half of the study (2006-2010), four of those patients (30.7%) had chemotherapy, compared to only 14.2% of patients (one of seven) between 2001 and 2005. Despite the differences in prior therapy over time, chemotherapy was not found to be a significant factor for successful closure, in this study.

## Discussion

With the evolution of organ sparing therapy for treatment of laryngeal and hypopharyngeal squamous cell carcinoma, chemoradiotherapy has increased over time. Thus, with laryngectomy becoming a salvage procedure, wound complications and pharyngocutaneous fistula have become an increasing concern. Wound healing complications can have a multifactorial origin including diabetes, hypothyroidism, radiation dosage, and chemotherapy [6,8,11]. Previous studies have confirmed the added benefit of vascularized tissue at the time of pharyngeal closure to reduce fistula and promote the return to oral diet [5,8,9]. In this study we review our reconstructive experience with patients who suffered pharyngocutaneous fistula after primary closure of a salvage laryngectomy wound. In our series of 20 patients we have demonstrated fistula closure to be a complex process in which only half of patients have definitive fistula closure with a single procedure. The current study supports the use of preplanned vascularized tissue use in the wound of the neopharynx after salvage laryngectomy.

**Figure 1:**
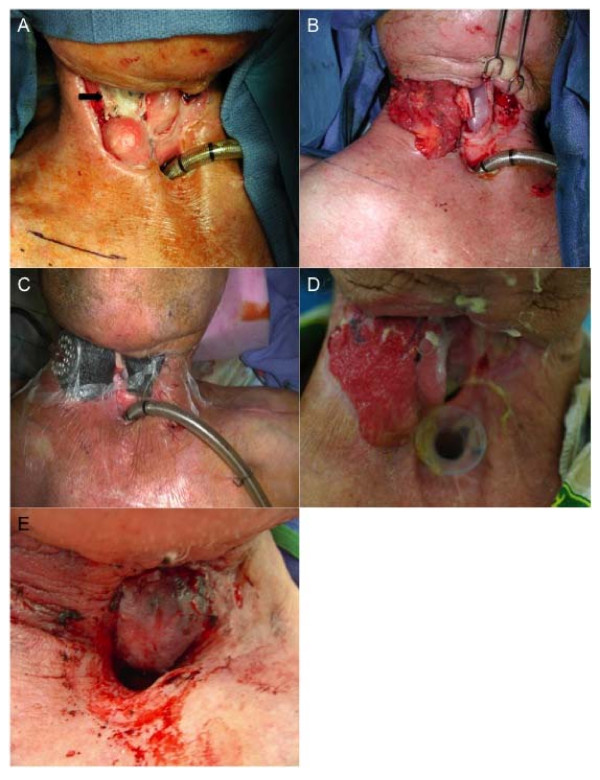
**Staged vessel protection, before pharyngocutaneous fistula closure using folded radial forearm free flap**. (A) Great vessels (arrow) bathed in saliva from adjacent pharyngocutaneous fistula, (B) with pectoralis pedicled flap the neck vessels are covered, (C) a negative pressure dressing facilitates wound care and controls the fistula in the immediate suprastomal area until (D) the wound is well granulated and (E) a folded radial forearm free flap is used to close the suprastomal fistula several weeks later.

It was elected to reconstruct most patients referred for fistula repair with radial forearm free flaps (RFFF), however the techniques of fistula closure varied with time and increasing experience. Each fistula required analysis of the amount of remaining mucosa, pliability and quantity of neck skin, and the presence of stricture to formulate a reconstructive plan. Patients with stricture-based fistula had the stricture incised and the free flap tissue inserted into the band of circumferential stricture, to allow passage of a 12 or 14 mm salivary bypass tube. When larger fistula (the classic three-hole defect) were closed using a single folded flap to reconstitute both the pharyngeal lining and the external skin, the vascular pedicle of the RFFF folded on itself and obstructed venous flow and as result led to refistulization. We found in the present study that patients who underwent a "patch" graft using the RFFF to reconstruct the pharyngeal lining, and then had good skin quantity and laxity to close the skin primarily overlying the free flap, had fewer leaks and required fewer additional procedures. Thus, in the patient population with minimal mucosa and skin available, we deduced over time that the combination of free flap patch closure with pedicled pectoralis muscle created the ideal reconstruction. The technique of folding the radial forearm skin paddle on itself to obtain closure of the internal and external lining did not routinely work well because of kinking the pedicle at the fold or re-fistulizing at the de-epithelialized portion of the skin paddle. An example of staged pectoralis muscle flap followed by successful folded RFFF is shown in Figure 1.

One flap failed in the series (4.5% failure rate). In this RFFF, the recipient vessels were the superior thyroid artery and external jugular vein. This flap was folded to allow the distal portion of the forearm to seal the pharynx and proximal paddle to close the neck. This patient required take-back on POD 2 and was salvaged only to require additional take-back on POD 3, complete venous clot was encountered, which was thought to be secondary the tight folding of the flap.

Rather we have favored the free flap to reconstitute the pharyngeal lining, while the pectoralis flap provides well-vascularized tissue as a substrate for skin grafting or granulating external wound closure. In patients who have already had attempts at closure via pectoralis flap, we have repositioned the pectoralis muscle to protect neck vasculature and relied on staged double free flaps to seal the neopharynx. Others have reported the benefit of using a single anterior lateral thigh flap with two skin paddles to perform internal and external epithelial lining [12]. It was noted in this study that earlier repair was less prone to complications [12]. In our patient series, the data did not support this finding. Most of our fistula patients presented for repair within the first six months (65%), and two of those 13 patients never achieved fistula closure. However, we did successfully close pharyngocutaneous fistula of more than 12 months duration in 3 patients without any complications.

**Figure 2:**
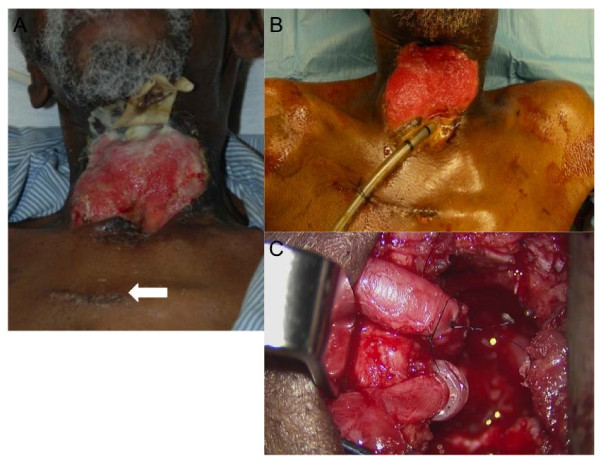
**Staged free flaps in closure of pharyngocutaneous fistula, after failure of primary closure in salvage laryngectomy**. (A) After tubed rectus free flap to divert fistula toward base of tongue region; arrow denotes internal mammary vessel incision site. (B) Prior to second stage, (C) with second set of internal mammary vessels, microvascular anastomosis was performed for patch-type radial forearm free flap in base of tongue region to achieve final closure.

As the closure technique has evolved over time, so has the selection of recipient vessels. In this patient series donor neck vessels were used 54.5%, most commonly the facial vessels, and internal mammary vessel were used in 45.5% of patients. Because neck exploration and vessel anastomosis was used in the two patients who developed carotid blowout, the use of cervical vessels in these patients was reconsidered. Both carotid blowout patients had previous neck dissection procedures, and chemoradiotherapy. The repetitive dissection of the chemoradiated neck with potential exposure to saliva for free flap vessel anastomosis was felt to significantly increase the risk for carotid blowout. To this end, the choice of recipient internal mammary vessels, as seen in Figure 2, allowed for free flap closure of the fistula without repeated dissection of neck vasculature in the field of radiation and previous surgical scarring. The combination of RFFF and internal mammary vessel is ideal because of the length of the vascular pedicle, the thin and generous quantity of skin, and the unradiated, donor vasculature. When the fistula is located suprastomally compared to in the base of tongue region there is generous length of the vascular pedicle. The use of transverse cervical vessels is also appropriate in this setting as described by others [12].

Interestingly, when the demographic factors were analyzed, the duration of the fistula prior to reconstruction did not impact the success of reconstruction. The length of time of the fistula being present probably does not increase the inflammatory and scarring factors in the wound bed, but more likely that the event of a fistula precipitates the inflammatory and scarring cascade to occur. Also, it was noted that untreated hypothyroidism was a significant comorbidity (p = 0.01) that predisposed to failure to close the fistula. This finding is also supported by previous studies [10,13]. Screening TSH levels should be a routine part of head and neck cancer surveillance. In patients who develop fistula after primary closure of a salvage larygectomy, the need for screening TSH is heightened. In our series of patients, when elevated TSH levels were noted, institution of thyroid hormone replacement and delay of free flap closure help to improve wound healing. For example, patient 11 (Table 1), presented with wound breakdown fistula after salvage laryngectomy, unsuccessful pectoralis pedicled flap closure, and a TSH level of 97.9. The patient underwent repositioning of the pectoralis muscle to protect the neck vasculature, thyroid hormone replacement therapy, and wound care. The first free flap stage was delayed several weeks to facilitate improved healing. A tubed rectus free flap was successfully performed, as all pharyngeal mucosa was lost, to intentionally divert saliva away from the stoma and contralateral neck vessels. Finally, four weeks after the first free flap, a RFFF was used to patch the superiorly based (base of tongue) fistula. Figure 2 demonstrates a patient with similar flap staging.

Recently published literature supports the use of vascularized tissue for decreased fistula in salvage laryngectomy wounds. Driven *et al. *found that patients undergoing salvage surgery within one year of completing concurrent chemoradiotherapy, or high dose radiotherapy (>64 Gray) were at increased risk for pharyngocutaneous fistula, 34.2% vs. 15.7% [5]. In 2007, Withrow *et al. *noted that use of vascularized free flap for laryngectomy closure had 18% fistula rate compared to 50% for primary closure [9]. Others have noted improvement in rate of pharyngocutaneous fistula after using pectoralis pedicled flaps for reinforcement of a primary laryngectomy closure after chemoradiation failure [8]. In our series of 20 patients we have demonstrated fistula closure to be a complex process in which only half of patients have definitive fistula closure with a single procedure. The current study supports the use of preplanned vascularized tissue use in the wound of the neopharynx after salvage laryngectomy. Although this may add time to an oncologic procedure, simultaneous planned use of pectoralis pedicled muscle or free flap tissue does decrease fistula and its associated complications [9].

## Conclusion

Patients that develop fistula after salvage laryngectomy with primary closure have a constellation of treatment and demographic factors that make reestablishing an oral diet a challenge. Choosing vascularized tissue to reconstitute both the pharyngeal lining and external skin in our case series is best done with radial forearm free flap and pedicled pectoralis muscle. Selecting donor vessels like the internal mammary site can avoid complications like carotid blowout. Planned preparation of vascularized tissue in a salvage laryngectomy neopharynx and control of comorbidities in the pre-operative period can minimize additional procedures and provide the best outcome for our salvage laryngectomy patients.

## Competing interests

The authors declare that they have no competing interests.

## Authors' contributions

**IA Bohannon **performed conceptual study design, data acquisition and interpretation, statistics, and drafting of manuscript. **WR Carroll **performed data interpretation, critical manuscript review. **JS Magnuson **performed data interpretation, critical manuscript review. **EL Rosenthal **performed conceptual study design, critical manuscript review. All authors have read and given approval to final manuscript.
